# Alpha-synuclein mutations impair axonal regeneration in models of Parkinson's disease

**DOI:** 10.3389/fnagi.2014.00239

**Published:** 2014-09-10

**Authors:** Lars Tönges, Éva M. Szegö, Patrizia Hause, Kim-Ann Saal, Lars Tatenhorst, Jan Christoph Koch, Zara d`Hedouville, Vivian Dambeck, Sebastian Kügler, Christoph P. Dohm, Mathias Bähr, Paul Lingor

**Affiliations:** ^1^Department of Neurology, University Medicine GöttingenGöttingen, Germany; ^2^Nanoscale Microscopy and Molecular Physiology of the Brain, Cluster of Excellence 171—DFG Research Center 103 (CNMPB)Göttingen, Germany; ^3^Department of Neurodegeneration and Restorative Research, University Medicine GöttingenGöttingen, Germany

**Keywords:** alpha-synuclein, A30P, A53T, 6-OHDA, axonal regeneration, dopaminergic cell death, GAP-43, ROCK

## Abstract

The dopaminergic (DAergic) nigrostriatal tract has an intrinsic regenerative capacity which can be impaired in Parkinson's disease (PD). Alpha-synuclein (aSyn) is a major pathogenic component in PD but its impact on DAergic axonal regeneration is largely unknown. In this study, we expressed pathogenic variants of human aSyn by means of recombinant adeno-associated viral vectors in experimental paradigms of DAergic regeneration. In a scratch lesion model *in vitro*, both aSyn(A30P) and aSyn(A53T) significantly reduced DAergic neurite regeneration and induced loss of TH-immunopositive cells while aSyn(WT) showed only minor cellular neurotoxic effects. The striatal density of TH-immunopositive axons in the striatal 6-OHDA lesion mouse model was attenuated only by aSyn(A30P). However, striatal expression levels of the regeneration marker GAP-43 in TH-immunopositive fibers were reduced by both aSyn(A30P) and aSyn(A53T), but not by aSyn(WT), which was associated with an activation of the ROCK signaling pathway. Nigral DAergic cell loss was only mildly enhanced by additional overexpression of aSyn variants. Our findings indicate that mutations of aSyn have a strong impact on the regenerative capacity of DAergic neurons, which may contribute to their pathogenic effects.

## Introduction

Since worldwide life expectancy increases, the numbers of individuals with Parkinson's disease (PD) will also strongly rise in the coming decades (Dorsey et al., 2007). In brains of PD patients, alpha-synuclein (aSyn) has been found in thread-like fibrils in Lewy body deposits inside dopaminergic (DAergic) neurons and also in dystrophic neurites (Spillantini et al., 1997; Del Tredici and Braak, 2012). Importantly, aSyn pathology does not only affect DAergic cells but has also been identified in other neuronal cell populations in the enteric nervous system, olfactory bulb, dorsal motor nucleus of the vagal and glossopharyngeal nerves and in other brain stem nuclei explaining the multitude of non-motor manifestations in PD (Postuma et al., 2012).

ASyn is implicated in familial genetic autosomal-dominant forms of PD. There exist whole-locus multiplications or point mutations of aSyn including the A53T and the A30P variant (Singleton et al., 2013). Whole-locus multiplications seem to lead to an earlier and more severe onset of disease depending on the gene copy number (Fuchs et al., 2007). The A53T variant has broadly varying phenotypes and is neuropathologically characterized by DAergic cell loss and a dense burden of aSyn neuritic pathology including axonal spheroids and rare Lewy bodies with fibrillar aSyn-immunoreactive aggregates (Duda et al., 2002). The first patho-anatomical study of an A30P mutation carrier showed DAergic cell loss in the substantia nigra (SN) as well as widespread occurrence of aSyn immunopositive Lewy bodies and Lewy neurites (Seidel et al., 2010). Here, the clinical symptoms are similar to idiopathic PD (Kruger et al., 2001). Several aSyn mutations have been transferred into animal models of disease by the means of genetically engineered transgenic mice or virus-mediated region-specific protein expression approaches (Low and Aebischer, 2012).

The initial pathology of PD is likely to be characterized by axonal degeneration, whereas the loss of DAergic neurons in the substantia nigra pars compacta (SNpc) follows subsequently (reviewed in Burke and O'Malley, 2013). In support of this hypothesis, the extent of terminal loss in the striatum seems to be much more prominent than the amount of cell loss of nigral DAergic neurons at the time when first clinical symptoms appear, suggesting that the striatal DAergic nerve terminals are the primary target of the degenerative process and that neuronal death in PD represents a “dying back” pathology (Dauer and Przedborski, 2003). The axonal insult can be modeled by striatal injection of 6-hydroxydopamine (6-OHDA) in mice and in rats. It causes an acute retrograde axonal degeneration followed by neuronal loss in the SN (Deumens et al., 2002). After lesion, the intrinsic regenerative potential of DAergic neurons is activated and it leads to a partial restoration of nigrostriatal tract fibers (Finkelstein et al., 2000).

In the current study we examined whether targeted expression of either human aSyn(WT) or the A30P and A53T variants in nigral DAergic cell populations affect neurite and axonal regeneration in lesioned midbrain DAergic neurons (MDN) *in vitro* and in 6-OHDA-lesioned DAergic nigral neurons *in vivo*. We found that the regenerative response is strongly impaired after expression of the mutated variants of human aSyn, the A30P variant being the most compromising one. Interestingly, the amount of striatal GAP-43-immunopositive regenerating structures was much more decreased than the density of TH-immunopositive fibers. Here, the impairment in regenerative growth was associated with an activation of the ROCK signaling pathway. The expression of aSyn(WT) resulted only in minor alterations. These findings indicate that genetic alterations of aSyn have a strong impact on the regenerative capacity of DAergic neurons.

## Materials and methods

### Recombinant vector production

AAV vectors were constructed to express human aSyn(WT), the A30P or A53T mutation as well as EGFP or only EGFP control. Viral vectors were prepared as previously described (Shevtsova et al., 2005). For *in vitro* applications, the AAV 1/2 or AAV6 serotype was chosen because of its increased transduction efficiency. In case of *in vivo* injections into the SN, the AAV2 serotype was selected because of its higher selectivity for neuronal cells. All AAV consisted of AAV ITRs, human synapsin-1 gene promoter driving expression of aSyn variants/EGFP or EGFP only, WPRE for enhanced mRNA stability and bovine growth hormone polyadenylation site (Taschenberger et al., 2012).

### *In vitro* DAergic cell culture and AAV vector application

Primary MDN were prepared according to previously published protocols (Knoferle et al., 2010). After the mesencephalon floor of embryonic day 14 Wistar rats had been dissected, the meninges were removed. Dissected tissue pieces were collected in ice-cold CMF and centrifuged at 1000 rpm for 4 min. Thereafter, Trypsin (750 μl, 0.25%, Sigma, Taufkirchen, Germany) was added to the tissue pellet and after 15 min incubation at 37°C, it was inactivated with 750 μl cold FCS. After gentle trituration of tissue fragments, the cell suspension was centrifuged at 1000 rpm for 4 min and resuspended in culture medium.

Cells were then plated at a density of 60,000/well in 96-well plates or at a density of 400,000/well in 24-well plates (Sarstedt, Nümbrecht, Germany) on coverslips. Coating was done with poly-D-lysine and laminin (DIV0). Four hours after plating, cultures were transduced with AAV for EGFP, human aSyn(WT) or the A30P or A53T aSyn mutations, each in a concentration of 1 × 10^8^ transducing units per well. Cell cultures were maintained at 37°C in a 5% CO_2_ humified atmosphere in DMEM-F12 (Invitrogen, Darmstadt, Germany) supplemented with 2.5 mg/ml BSA (35%), 0.9% D-(+)-glucose solution (45%), 2 mM L-glutamine (PAA Laboratories, Pasching, Austria), 5 μg/ml insulin, 1:100 N1 medium supplement and 1:100 PSN antibiotic mixture (Invitrogen, Scotland, UK). On the next day (DIV1) half of the medium was replaced. On DIV3 cells in 24 well-plates on coverslips were submitted to mechanical transection using a self-made 2 mm broad silicon rubber scratch device taking care not to damage the poly-D-lysine and laminin coating (Tönges et al., 2011b). After another three days on DIV6, cells were processed for immunocytochemistry or for cell viability and toxicity assays.

### *In vitro* immunocytochemistry, quantification of neuronal survival and neurite regeneration

To perform immunocytochemistry, cells plated on coverslips were fixed in PFA 4% for 10 min at room temperature (RT, 22°C), washed twice with PBS, incubated 30 min in 25 mM Glycine in PBS (Applichem, Darmstadt, Germany), permeabilized and blocked with 10% horse serum, 5% BSA, 0.3% Triton, 25 mM Glycine in PBS at RT for 1 h. Probes were incubated with mouse anti-tyrosine hydroxylase 1:500 (Sigma, St. Louis, MO, USA), rabbit anti-tyrosine hydroxylase 1:1000 (Zytomed Systems, Berlin, Germany), goat anti-tyrosine hydroxylase 1:500 (abcam, Cambridge, UK), chicken anti-GFP 1:500 (abcam, Cambridge, UK), mouse anti-human aSyn LB509 1:1000 (Covance, Princeton, NJ, USA) overnight at 4°C in blocking solution. Following three PBS washes, the respective secondary antibodies [all 1:500, donkey anti-mouse Alexa Fluor 488 (Life Technologies, Darmstadt, Germany), donkey anti-goat Cy5, donkey anti-goat Cy3, donkey anti-rabbit Cy3, goat anti-mouse Cy2, goat anti-mouse Cy3, goat anti-rabbit Cy3 (Dianova, Hamburg, Germany), goat anti-mouse Cy5, goat anti-rabbit Cy5, goat anti-chicken Alexa Fluor 488 (Jackson, Suffolk, UK)] were applied for 1 h at RT. Cells were then nuclear counter-stained with DAPI (4,6-diamidino-2-phenylindole) (Sigma, St. Louis, MO, USA) and mounted in Mowiol (Hoechst, Frankfurt, Germany).

In order to quantify DAergic cell survival in culture and neurite regeneration from the scratch site, microscopic pictures were taken with a Zeiss Axioplan 2 fluorescence microscope equipped with a CCD camera and AxioVision software (Zeiss, Göttingen, Germany). For evaluation of DAergic survival within the culture, pictures of five random visual fields per coverslip at least 200 μm apart from the scratch border were taken with a 20× objective and numbers of TH-immunopositive neurons were counted. Three samples of at least two separate experiments were quantified for each condition. The quantification of TH-immunopositive regenerating neurites was done semi-automatically with help of the NeuronJ axon tracing module of Image J. The length of the 10 longest axons extending from the scratch site of each photomicrograph (10× objective) was measured. Three samples of at least two separate experiments were quantified for each condition.

For the analysis of subcellular aSyn distribution, MDN transduced with the different AAV and immunostained against human aSyn (LB509, Covance), EGFP and TH were analyzed with a Leica SP5 confocal microscope (Leica, Biberach, Germany). Micrographs of TH-positive neurons were taken at 63× magnification with an additional 6× zoom factor. Quantification of aSyn fluorescence intensity was performed with Image J (Free Java software provided by the National Institutes of Health, Bethesda, Maryland, USA). The fluorescence intensity was measured in a region of interest (ROI) comprising the largest part of the soma and in a principal neurite at a distance of 50 μm from the soma over a length of 10 μm along the neurite. After subtraction of the background signal, the integrated density values given by Image J for each ROI were used as single fluorescence intensity values to calculate the ratio soma/neurite (*n* = 20 neurons per condition, 2 independent MDN cultures).

### Cell viability and toxicity assays

Cell viability and toxicity assays were performed in 96-well plates after 6 days in culture. To determine cell viability overall metabolic activity in culture was quantified using the WST1 assay (Roche, Mannheim, Germany) according to the manufacturer's instructions. Briefly, WST1 reagent was added 1:10 to the culture medium and incubated for 2 h at 37°C. Absorbance was measured at 450 against 620-nm reference wavelength using an ELISA reader (Tecan). To determine cell toxicity, a bioluminescence-based assay for the release of adenylate kinase (AK) from lesioned cells was applied according to the manufacturer's instructions (ToxiLight®, Lonza, Wakersville, USA). Briefly, the amount of AK was determined in the culture medium by measuring the AK-dependent conversion of ADP to ATP and subsequent light emission by luciferase with a luminometer (Wallac 1450 MicroBeta Trilux, PerkinElmer, Shelton, USA).

### Immunoblotting

Primary MDN cultures were plated at a density of 400,000/well in 24-well plates. Four hours after plating, cultures were transduced with AAV for EGFP, human aSyn(WT), the A30P or A53T aSyn mutations, each in a concentration of 1 × 10^8^ transducing units per well. After 6 days cells were lysed with a cell lysis buffer (Thermo Scientific™ Pierce™ RIPA Buffer, Thermo Fisher Scientific Inc., Waltham, MA, USA) plus protease inhibitors (“Complete tablets,” Roche, Basel, Switzerland) and phosphatase inhibitor (“PhosSTOP,” Roche, Basel, Switzerland). The protein content of all cell samples was determined using Bradford (Biorad, Munich, Germany) and equal amounts of protein (20 μg) were separated on a sodium dodecyl sulfate–polyacrylamide gel electrophoresis. Proteins were then electrotransferred onto a PVDF membrane (Applichem, Darmstadt, Germany) and blocked with 5% milk in Tris-buffered saline/Tween-20 (TBS-T) for 1 h. Membranes were then incubated with the primary antibodies anti-human aSyn 1:500 (Invitrogen, Carlsbad, CA, USA), anti-MYPT1 1:500 (SCBT, Dallas, USA) and anti-β Tubulin 1:10000 (Sigma, St. Louis, MO, USA) in 5% milk TBS-T and anti-phospho-MYPT1 1:500 (Merck Millipore, Darmstadt, Germany) in 5% BSA TBS-T over night at 4°C. This was followed by incubation with corresponding horseradish peroxidase-coupled secondary antibodies (1:1000 for 1 h at room temperature; Dianova, Hamburg, Germany). ECL-Plus reagent (Amersham, Arlington Heights, IL, USA) was applied on the membrane and the chemiluminescence was visualized on an Amersham Hyperfilm ECL (GE Healthcare, Chalfont St Giles, GB).

### *In vivo* 6-OHDA lesion and AAV vector application

All experimental animal procedures were conducted according to approved experimental animal licenses issued by the responsible animal welfare authority (Niedersächsisches Landesamt für Verbraucherschutz und Lebensmittelsicherheit) and controlled by the local animal welfare committee of the University Medical Center Göttingen. Animals were housed separately in individually ventilated cages (IVC, Tecniplast, Hohenpeißenberg, Germany) and were kept under a 12 h light/12 h dark cycle with free access to food and water.

Stereotactical injections of 6-OHDA (Sigma) were performed as described before (Alvarez-Fischer et al., 2008). Briefly, 8–10 weeks-old male C57Bl/6 mice were anesthetized with ketamine (200 mg/kg) and xylazine (10 mg/kg). The unilateral right striatum was lesioned by stereotactic injection of 6-OHDA [4 μg, dissolved in a volume of 2 μl 0.2% L-ascorbic acid (L-AA, Sigma) in PBS] or vehicle (2 μl, 0.2% L-AA in PBS) at an injection rate of 0.5 μl/min using the following coordinates: AP: +0.04 cm, ML: −0.18 cm, DV: −0.35 cm. After injection the needle was left in place for 5 min before removal. In case of additional AAV application, the animals received a stereotaxic injection into the SN directly after 6-OHDA lesion (AP: −0.29 cm, ML: −0.13 cm, DV: −0.45 cm) with AAV2 expressing EGFP, human aSyn(WT) or the A30P or A53T aSyn mutations, each with a total of 1 × 10^8^ transducing units dissolved in 1 μl PBS at an injection rate of 0.5 μl/min (Suppl. Fig. 1).

### Immunohistochemistry and quantification of DAergic neuronal loss and axonal regeneration

Mice were sacrificed by exposure to carbon dioxide 4, 8, or 12 weeks after striatal 6-OHDA lesion and, in the case of the 12 weeks experiment, additional AAV injection into the right SN. This was followed by a transcardial perfusion with PBS and later with 4% paraformaldehyde (PFA) in PBS (pH 7.4). Brains were dissected, post-fixed in PFA overnight at 4°C, cryoprotected in 30% sucrose in PBS for 24 h at 4°C, snap frozen, and stored at −80°C until sectioning. For each group frontal cryosections (30 μm) through the SN and the striatum were collected and kept in 0.1% sodium azide/PBS. The further processing employed a free floating method of immunohistochemistry.

In order to label TH-immunopositive cells in the SNpc, sections were rinsed in 0.1 M TBS (pH 7,4) followed by a treatment of 10% methanol and of 3% H_2_O_2_ in TBS for 5 min, then rinsed again for three times in TBS and blocked with 5% normal goat serum (NGS) in TBS for 60 min. Next, sections were exposed to a rabbit anti-tyrosine hydroxylase antibody (1:1000; Zytomed, Berlin, Germany) in 1% NGS for 48 h at 4°C. After another three TBS wash steps the sections were exposed to a secondary biotinylated antibody (1:200, Dianova, Hamburg, Germany) in TBS at RT for 60 min followed by rinsing in TBS again and incubation with the VECTASTAIN ABC Peroxidase (standard Kit, PK-4000, Biozol, Eching, Germany) for 60 min at RT. After another TBS wash step the sections were treated with 3,3′-diaminobenzidine (DAB peroxidase substrate Kit, SK-4100, Vector Laboratories, Germany) for 4 min, rinsed again and collected on object slides. Ultimately, sections were mounted with DPX (Fluka Chemica, Switzerland).

In order to evaluate the density of TH-immunopositive striatal fibers, a protocol was applied as published previously (Schulz et al., 1995). Briefly, slices were incubated in chloroform–ethanol followed by dehydration, use of anti-tyrosine hydroxylase antibody (1:500, Zytomed, Berlin, Germany) and DAB staining (Elite ABC Kit, Vector Laboratories), nickel intensification, formalin fixation and dehydration. Entellan® was used to mount the sections.

The detection of GAP-43-immunopositive staining in TH-immunopositive fibers in the striatum was performed with a fluorescence-based labeling method. Here, brain slices were incubated in 1% H_2_O_2_/40% methanol for 15 min, then in 1 mM EDTA (pH 8) at 80°C for 30 min. Primary anti-GAP-43 (1:1000, Millipore) and anti-tyrosine hydroxylase antibody (1:500, Zytomed, Berlin, Germany) were used together with the respective secondary antibodies (1:2000, Alexa Fluor 488 or Alexa Fluor 594, Life Technologies, Darmstadt, Germany) to visualize GAP-43 and TH signal.

Quantification of DAergic cell numbers in the SNpc was done by counting TH-positive neurons using the Stereo Investigator software (Stereo Investigator 9.0, MicroBrightField Inc., Colchester, VT, USA, Zeiss microscope; Dehmer et al., 2004). Here, every fourth nigral section was evaluated by overlaying a point grid and counting of TH-immunolabeled cells with the optical fractionator method (40× objective). Values represent counts in the unilateral SN of the hemisphere with striatal 6-OHDA lesion or with striatal vehicle control injection.

Quantification of striatal TH-density was done as described earlier (Szegö et al., 2012). Every fourth section was stained and six sections were analyzed per animal. Six images were taken of every slice using a 63× objective (Axioskop 2, Zeiss, Jena, Germany) and the area covered by TH-immunopositive fibers was measured ten times from the same image following background subtraction and binarization. Therefore, six sections per animal, six images per section and 10 measurements per image resulted in a hierarchically nested design. A generalized linear mixed model was applied. The identification numbers of animals, sections and images were set as random factors (R software; R Development Core Team).

For the quantification of the colocalization of GAP-43 and TH signals, every fourth section was stained with fluorescent dyes. Images were taken using a 100× objective (Olympus IX51, Olympus Germany, Hamburg, Germany). For analysis, the area of specific staining was measured using ImageJ colocalization plug-in.

### Statistical analysis

Statistical multiple group comparisons were conducted by One-Way ANOVA followed by Tukey's *post-hoc* test. Striatal fiber density measurements were performed in a hierarchically nested design (Szegö et al., 2012). For statistical analyses, Kyplot software version 2.0 (KyensLab Incorporated) and R software version 2.8.0 (R Development Core Team) were used. Data are represented as mean ± s.e.m. as indicated. Significances are indicated with ^*^p < 0.05; ^**^p < 0.01; ^***^p < 0.001.

## Results

### Survival of TH-immunopositive neurons is reduced after AAV-mediated expression of aSyn

**Figure 1:**
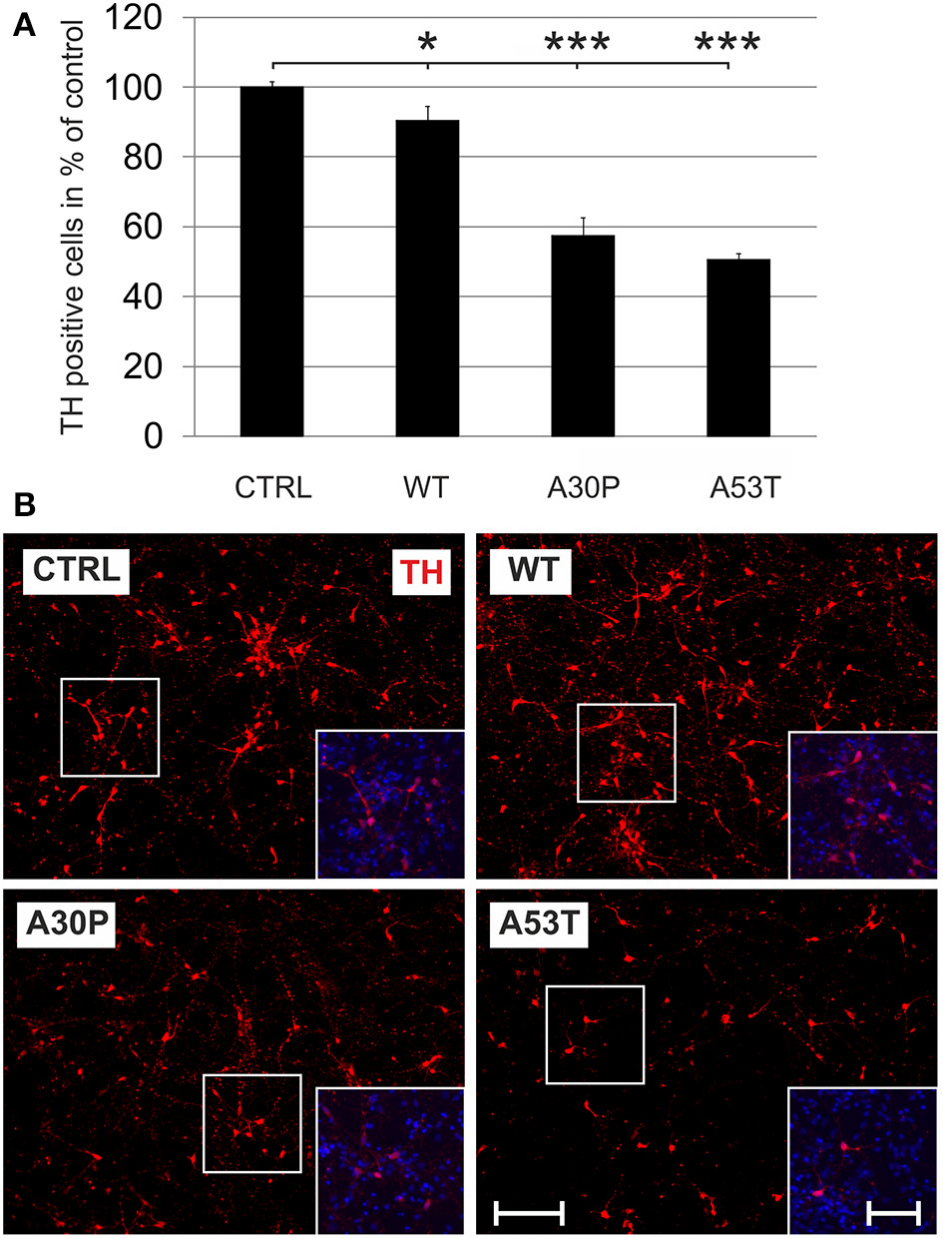
**Neurotoxicity of AAV-mediated aSyn expression in MDN culture**. Neurons were transduced 4 h after plating with AAV vectors expressing EGFP (CTRL) or the aSyn variants (1 × 10^8^ transducing units/400,000 neurons/well) and were evaluated for cell survival after 6 days in culture. **(A)** Histogram showing percentages of surviving tyrosine hydroxylase (TH)-immunopositive neurons in comparison to the EGFP control. Bars represent means ± s.e.m. ^*^p < 0.05, ^***^p < 0.001. **(B)** Representative micrographs of MDN cultures labeled against TH (Cy3, red). Insets show additional DAPI nuclear counter-staining (blue). Scale bar = 400 μm, scale bar inset = 200 μm.

Adeno-associated viral vectors were generated expressing human aSyn(WT), mutated human aSyn(A30P), or aSyn(A53T) as well as EGFP under the human synapsin I promoter. A vector which expressed only EGPF under the human synapsin I promoter was used as control. All constructs led to a comparable transduction efficiency as visualized by their EGFP fluorescence. The expression of human aSyn was similar for all human aSyn-expressing AAV (Suppl. Fig. 2). In order to evaluate if expression of aSyn leads to death of DAergic cells *in vitro*, we transduced MDN cultures with the respective AAV with a titer of 1 × 10^8^ transducing units/400,000 cells shortly after plating and quantified TH-immunopositive cell numbers after 6 days. Here, minor DAergic cell loss was observed in the aSyn(WT) group (90 ± 4%) if compared to the EGFP control (100 ± 2%). However, cell survival was much more reduced in aSyn(A30P) and aSyn(A53T) transduced cultures (A30P: 57 ± 5%; A53T: 51 ± 2%), suggesting that both mutated aSyn exerted a higher neurotoxicity to DAergic cells than the wildtype form (Figure 1). Numbers of TH-negative cells were unchanged after application of the different AAV as evaluated on DIV6 (Supplementary Table 1). The evaluation of metabolic activity and of cytotoxicity-related release of AK in MDN cultures showed an impairment in all aSyn infected cultures in comparison to the EGFP control, however, without exhibiting differences between the various forms of aSyn (Suppl. Fig. 3).

### Regeneration of scratch-lesioned TH-immunopositive neurites is decreased after AAV-mediated expression of mutated aSyn

**Figure 2:**
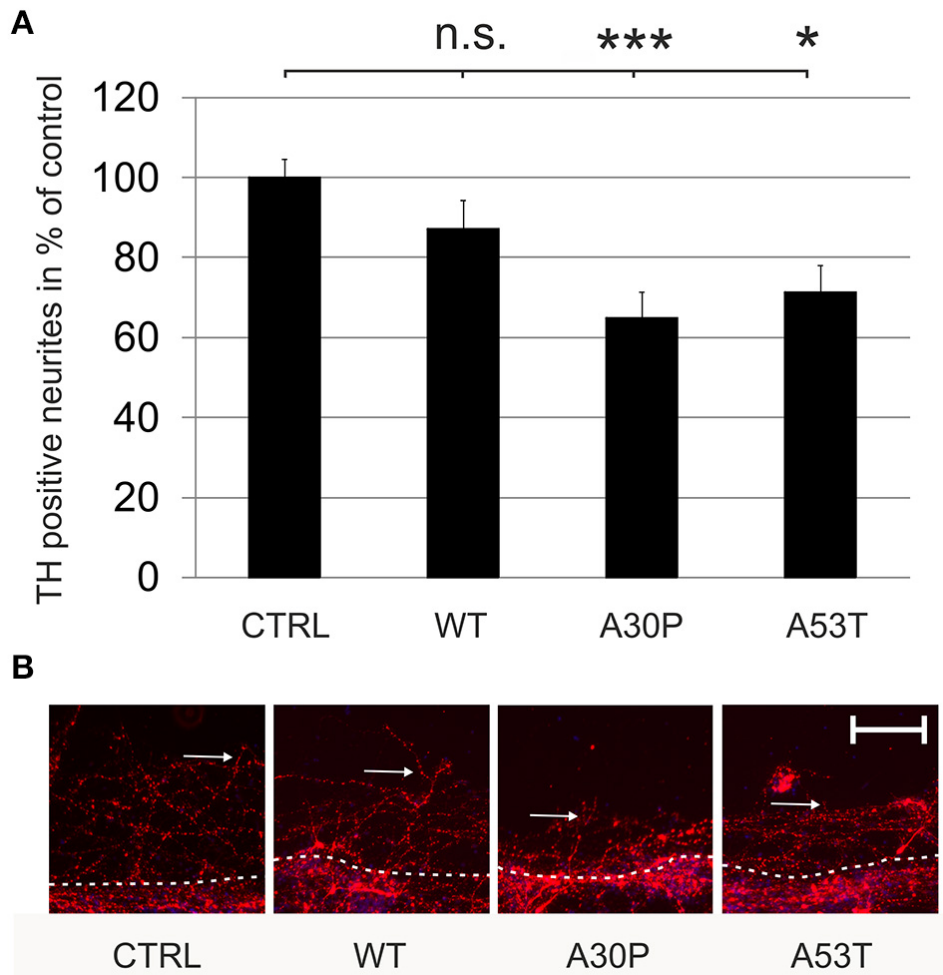
**Regeneration of TH-immunopositive neurons is decreased by AAV-mediated expression of mutated aSyn**. Neurons were transduced 4 h after plating with the respective AAV vectors expressing EGFP (CTRL) or the aSyn forms (1 × 10^8^ transducing units/400,000 neurons/well) and were scratch lesioned after 3 days in culture. Neurite regeneration was evaluated 3 days after scratch lesion. **(A)** Histogram showing the relative outgrowth of TH-immunopositive neurites in comparison to the EGFP transduced control. Bars represent means ± s.e.m. n.s. = not significant; ^*^p < 0.05. **(B)** Representative micrographs of MDN cultures from the site of scratch lesion labeled against TH (Cy3, red). *Dashed line*, site of mechanical transection (scratch lesion); *arrows*, distal end of regenerating neurites. Scale bar = 100 μm. ^***^p < 0.001.

Mutated aSyn is known to contribute to axonal pathology of DAergic cells. However, it is not known if aSyn or its mutated forms impair axonal regeneration. After the transduction of MDN with aSyn expression plasmids encapsuled in AAV vectors we performed a mechanical neurite scratch lesion and quantified the length of regenerating neurites after three more days in culture. We did not observe a significant difference in neurite regeneration in cell cultures, which had been transduced with wildtype aSyn (87 ± 7%) or the EGFP control (100 ± 5%). In contrast, transduction of the mutated forms of aSyn both resulted in a strong decrease of neurite regeneration which was most pronounced for the A30P variant [A30P (65 ± 6%); A53T (71 ± 7%)] (Figure 2). This implies that the aSyn mutations both interfere with DAergic neurite regenerative behavior *in vitro*. Numbers of TH-immunopositive cells at the first 100 μm of the scratch border were comparable to data obtained for the survival of DAergic neurons in culture as demonstrated before (Supplementary Table 2).

**Figure 3:**
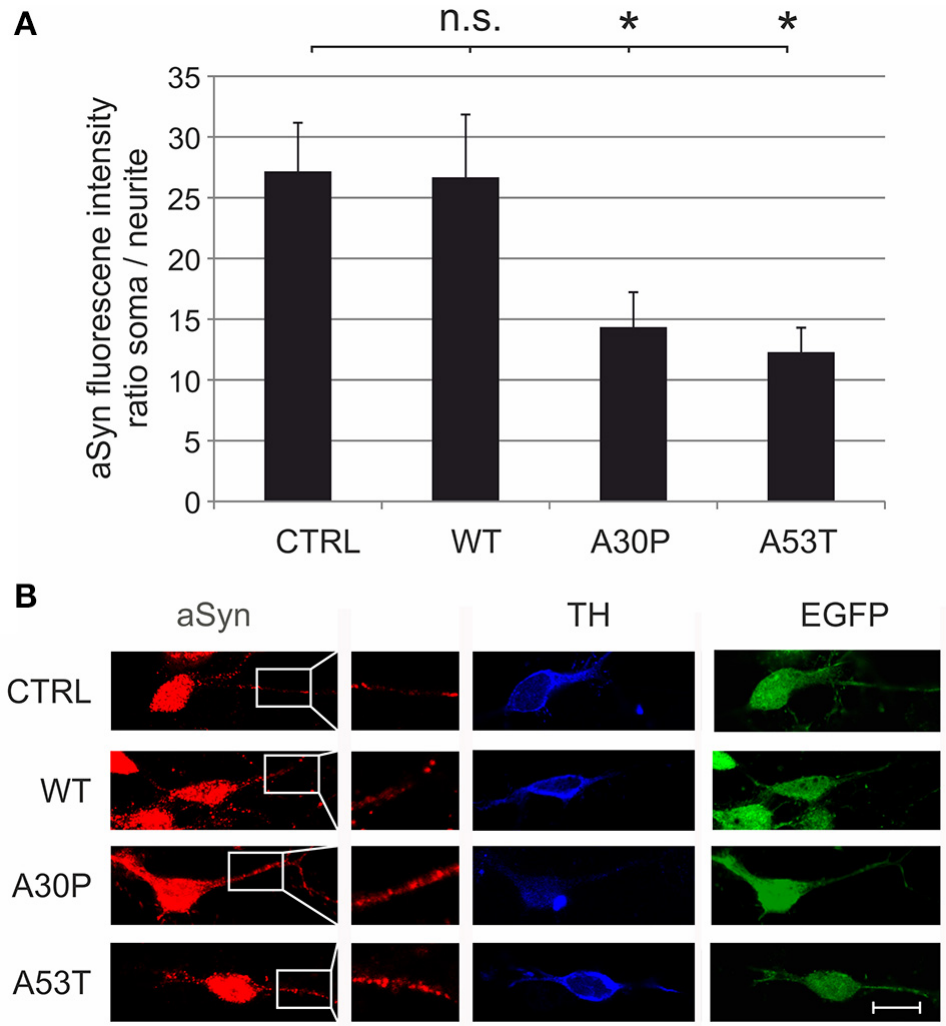
**Subcellular distribution of aSyn fluorescence intensity in MDN transduced with aSyn WT and mutant forms**. MDN transduced with AAV vectors expressing EGFP (CTRL) or the given aSyn forms (1 × 10^8^ transducing units/400,000 neurons/well) were immunostained against aSyn and TH. The fluorescence intensity of aSyn was quantified in the soma and in the neurite and the respective ratio was calculated **(A)**. Bars represent means ± s.e.m. n.s. = not significant; ^*^p < 0.05. **(B)** Representative confocal micrographs of AAV-transduced (EGFP-positive, green) dopaminergic (TH-positive, Cy5, blue) neurons stained against aSyn (Cy3, red). Scale bar = 30 μm.

These effects might be caused by increased local concentrations of aSyn in the neurites that impair neurite outgrowth. To test this hypothesis, we quantified its fluorescence intensity on confocal micrographs of MDN transduced with the different AAV vectors, which had been immunostained against aSyn and TH. When we calculated the ratio between the fluorescence intensity in the soma and in the neurites of TH-positive neurons, we found that this ratio was significantly decreased in MDN expressing A30P (14 ± 3) and A53T (12 ± 2) but not WT (27 ± 5) as compared to EGFP control (27 ± 4) (Figure 3). These data strongly suggest an accumulation of aSyn in the neurites of TH-positive neurons transduced with the mutant forms aSyn(A30P) and aSyn(A53T).

### DAergic cell survival in the SN after striatal 6-OHDA lesion is not significantly influenced by aSyn overexpression

**Figure 4:**
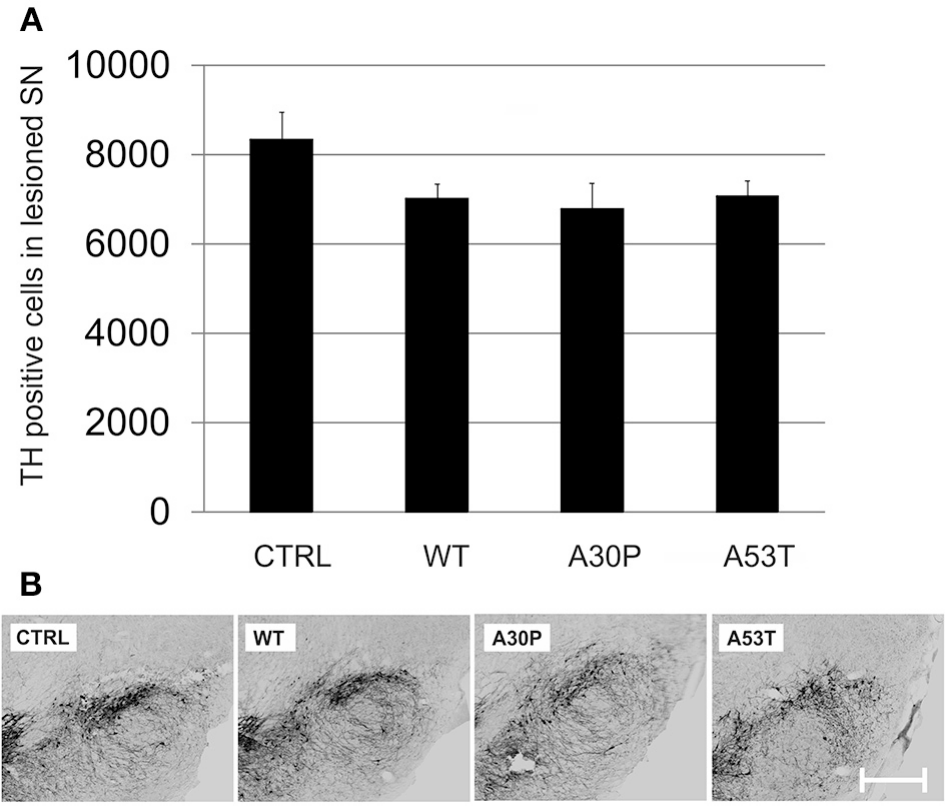
**DAergic survival in the SNpc 12 weeks after AAV-mediated nigral aSyn expression and ipsilateral striatal 6-OHDA lesion**. Nigral neurons were unilaterally transduced with AAV vectors expressing EGFP (CTRL) or aSyn (WT, A30P and A53T form; each 1 × 10^8^ transducing units) and were simultaneously lesioned in the ipsilateral striatum with 6-OHDA (4 μg/2 μl). **(A)** Histogram showing numbers of surviving TH-immunopositive nigral neurons 12 weeks after lesion and AAV injection. Bars represent means ± s.e.m. **(B)** Representative micrographs of substantia nigra mouse brain sections labeled against TH (black). Scale bar = 500 μm.

We then employed the 6-OHDA-mouse model of PD to test our hypothesis that aSyn might interfere with DAergic cell survival and more importantly with long-term axonal regeneration *in vivo*. First, the lesioning effects of unilateral stereotactically guided striatal 6-OHDA injections in mice on nigral DAergic cell loss were evaluated. We observed only a moderate loss of DAergic neurons in the unilateral SNpc, which persisted from 4 to 12 weeks after striatal lesion (Suppl. Fig. 4). The additional ipsilateral nigral injection of AAV vectors with plasmids expressing human aSyn(WT) or its mutants resulted in a slightly more pronounced loss of DAergic cells 12 weeks after transduction and 6-OHDA lesion. However, the DAergic cell loss was not significant for either aSyn(WT) (7021 ± 330 cells) or its mutants A30P (6783 ± 580 cells) and A53T (7069 ± 353 cells) in comparison to the EGFP control (8328 ± 618 cells) (Figure 4). Thus, there was only modest additional perikaryal neurotoxicity induced by aSyn overexpression in the SNpc.

### Mutated aSyn impairs striatal DAergic fiber regeneration after 6-OHDA lesion

**Figure 5:**
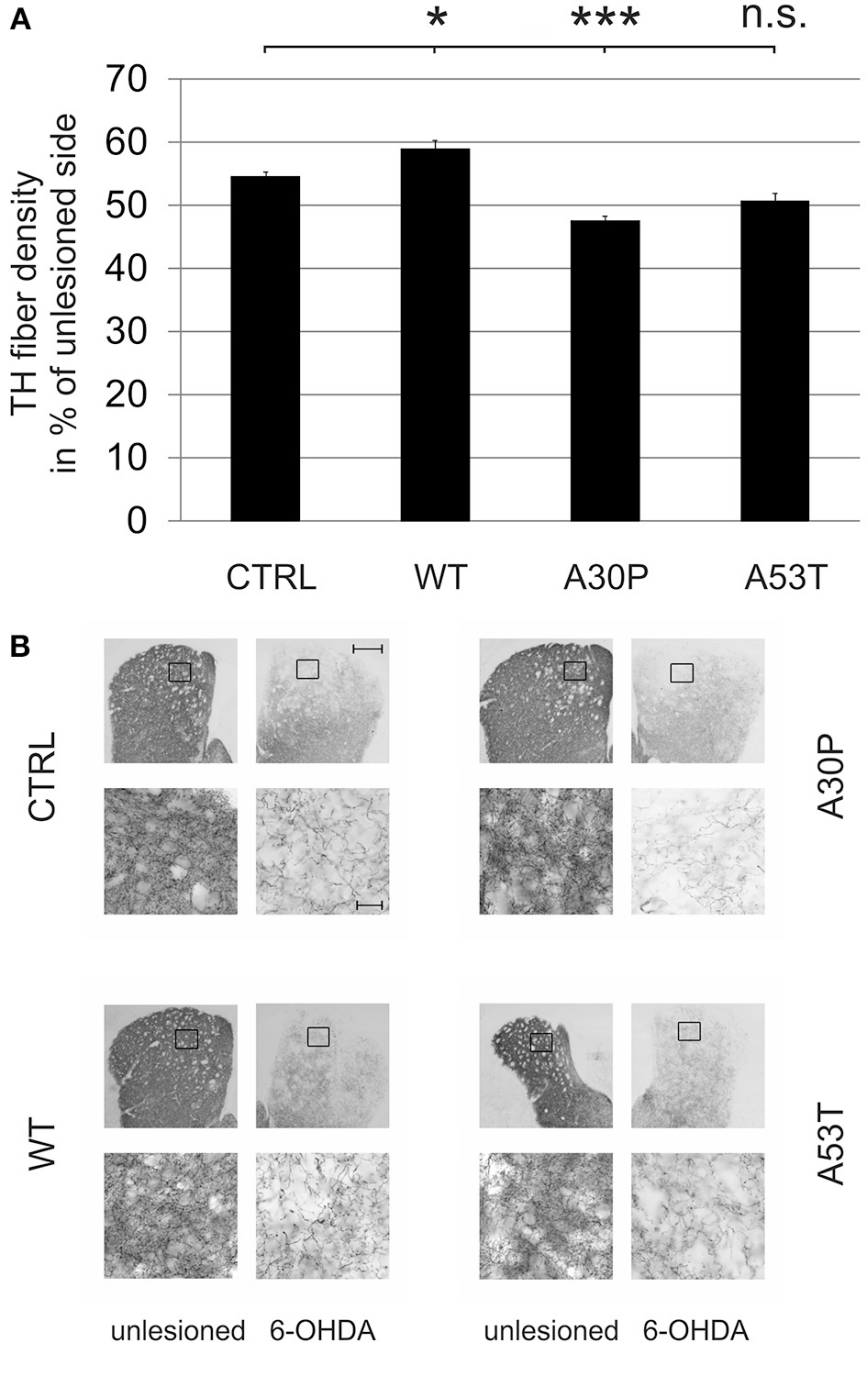
**Density of TH-immunopositive striatal fibers 12 weeks after AAV-mediated nigral aSyn expression and ipsilateral striatal 6-OHDA lesion**. Nigral neurons were unilaterally infected with AAV vectors expressing EGFP (CTRL) or aSyn (WT, A30P and A53T form; each 1 × 10^8^ transducing units) and were simultaneously lesioned in the ipsilateral striatum with 6-OHDA (4 μg/2 μl). **(A)** Histogram showing the relative density of TH-immunopositive striatal fibers in comparison to the unlesioned striatum 12 weeks after lesion and AAV vector injection. Bars represent means ± s.e.m. n.s. = not significant; ^*^p < 0.05, ^***^p < 0.001. **(B)** Representative micrographs of the striatum and magnifications after immunostaining for TH (black). Black squares mark exemplary fields of fiber analysis. Scale bar = 500 μm (overview), 20 μm (magnification).

In order to examine an impact of aSyn on axonal regeneration *in vivo* we quantified the density of TH-immunopositive fibers in the striatum after unilateral striatal 6-OHDA lesion and AAV-mediated ipsilateral nigral expression of aSyn. Establishing this model system we observed a severe loss of striatal TH-fibers 4 weeks after local 6-OHDA lesion, which recovered after 8 weeks. After 12 weeks, the fiber density had increased up to 60% of the unlesioned side (Suppl. Fig. 5). The additional AAV-mediated expression of aSyn(WT) or its mutants in the ipsilateral SNpc influenced the relative striatal TH-immunopositive fiber density compared to the unlesioned side 12 weeks after transduction. Whereas aSyn(A30P) (47.4 ± 0.8%) significantly reduced the fiber density if compared to EGFP (54.5 ± 0.8%), aSyn(A53T) reduced the density of TH fibers only by trend (50.6 ± 1.3%). The expression of aSyn(WT) resulted in a small increase of the TH fiber density (58.8 ± 1.4%) (Figure 5).

**Figure 6:**
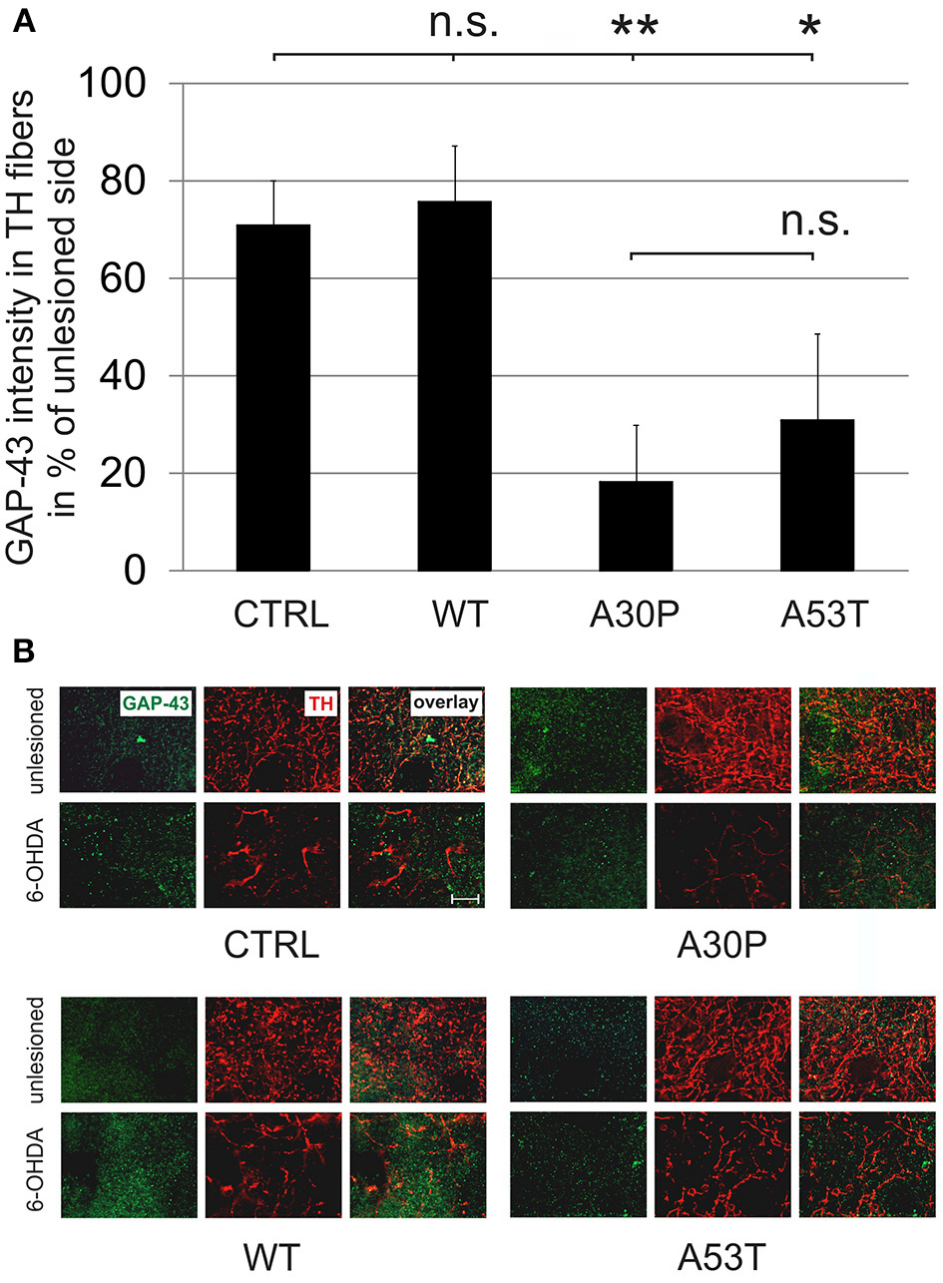
**Density of GAP-43 protein expression in TH-immunopositive striatal fibers 12 weeks after AAV-mediated nigral aSyn expression and unilateral striatal 6-OHDA lesion**. Nigral neurons were unilaterally infected with AAV vectors expressing EGFP (CTRL) or aSyn (WT, A30P and A53T form; each 1 × 10^8^ transducing units) and were simultaneously lesioned in the unilateral striatum with 6-OHDA (4 μg/2 μl). **(A)** Histogram showing the relative intensity of GAP-43 in TH-immunopositive striatal structures in comparison to the unlesioned striatum 12 weeks after lesion and AAV injection. Bars represent means ± s.e.m. n.s. = not significant; ^*^p < 0.05, ^**^p < 0.01. **(B)** Representative micrographs of the striatum in high resolution after immunostaining for GAP-43 (green) and TH (red). Scale bar = 20 μm.

**Figure 7:**
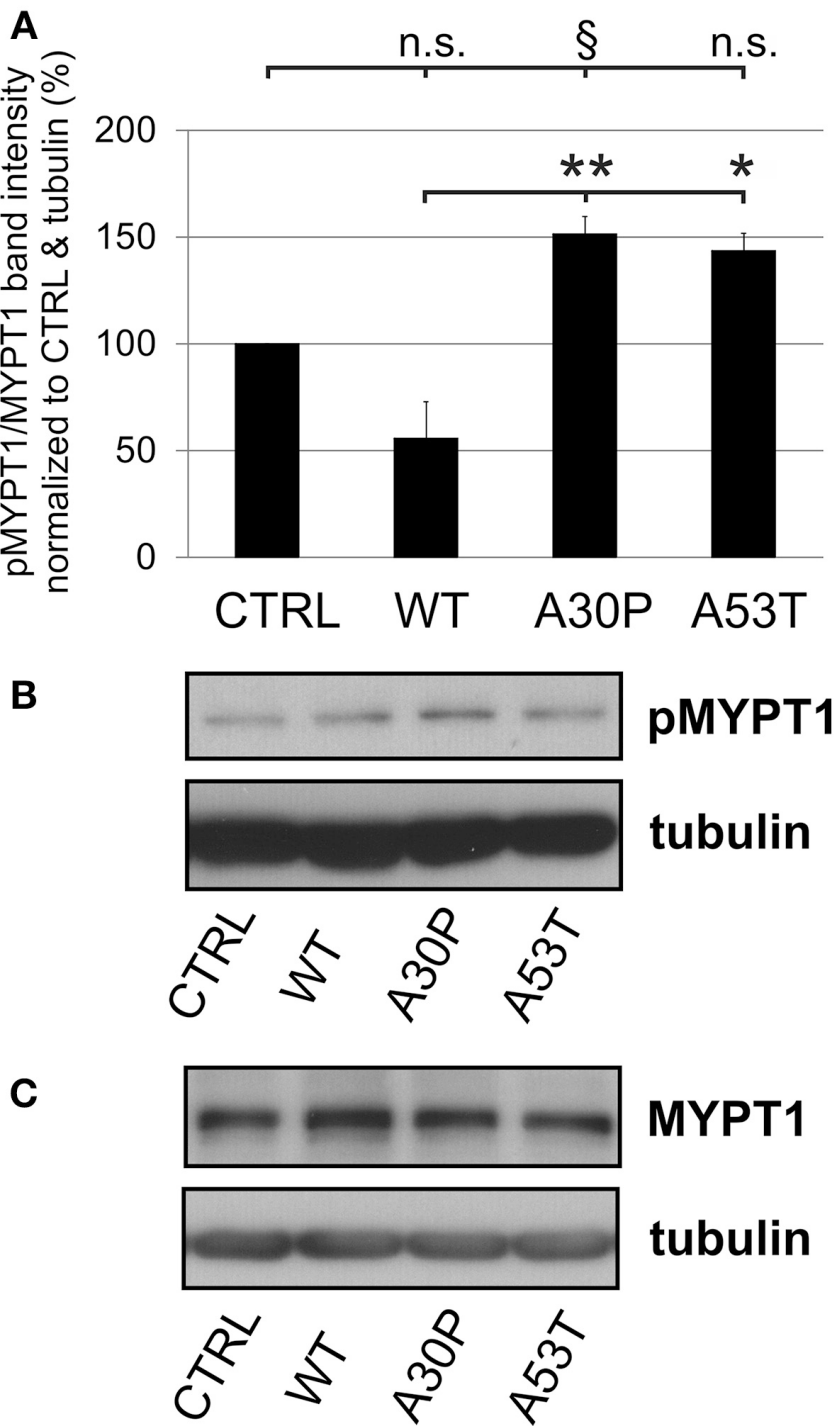
**Activation of ROCK downstream targets by human aSyn mutants**. **(A)** Quantification of pMYPT/MYPT ratio normalized to CTRL and tubulin in MDN cultures infected with AAV constructs (each 1 × 10^8^ transducing units) expressing EGFP (CTRL) or variants of aSyn (WT, A30P, or A53T) in MDN after 6 days in culture. Bars represent means ± s.e.m. n.s. = not significant; ^*^p < 0.05, ^**^p < 0.01, ^§^*p* = 0.08. **(B)** Representative immunoblots of pMYPT1 and tubulin. **(C)** Representative immunoblots of total MYPT1 and tubulin.

The quantification of striatal TH-immunopositive fiber density after 6-OHDA lesion is only an indirect measure of regenerating fibers. Therefore, we decided to examine the presence of GAP-43 in the lesioned striatum as a more specific marker for axonal regeneration. The quantification of GAP-43 expression in DAergic TH-immunopositive fibers of the nigrostriatal tract showed a reduction of GAP-43 protein levels in the lesioned striatum as compared to the unlesioned side in control animals, which had been transduced with an EGFP-expressing plasmid (70.8 ± 9.2%). In case of AAV-mediated aSyn(WT) expression, GAP-43 expression amounted to similar levels (75.8 ± 11.5%). However, the nigral expression of aSyn(A30P) resulted in significantly lower GAP-43 levels (18.2 ± 11.7%), which was also the case, although to a lesser extent, for aSyn(A53T) (30.9 ± 17.9%) (Figure 6). This implies that nigral overexpression of both aSyn(A30P) and aSyn(A53T) markedly impair the regenerative response of nigrostriatal tract DAergic neurons. As a putative mechanism we have identified the involvement of the rho kinase (ROCK) pathway. After overexpression of both aSyn(A30P) and aSyn(A53T) in MDN the ROCK downstream target MYPT1 was clearly phosphorylated indicative of an activation of this pathway. Interestingly, aSyn(WT) did not activate but rather attenuate ROCK downstream signaling being in line with its stabilization of GAP-43 levels (Figure 7).

## Discussion

Axonal degeneration is one of the most striking neuropathological characteristics of PD, which contributes to the progression of clinical symptoms (Burke and O'Malley, 2013). The presence of excess wildtype or mutated aSyn is known to amplify axonal damage and may additionally lead to cell death of DAergic neurons (Kirik et al., 2002; Lo Bianco et al., 2002). However, it is not known to which extent aSyn or its mutations further increase DAergic cell loss and axonal degeneration *in vivo* in the presence of additional stress factors such as 6-OHDA. More importantly, the impact on the intrinsic regenerative capacity of DAergic fibers by aSyn is only insufficiently characterized. Therefore, we have designed appropriate model systems to delineate the role of aSyn and its mutants in this context. We have found that the A30P and the A53T mutant forms of aSyn impair neurite and axonal regeneration most severely, whereas the impact on perikaryal cell death is only moderate.

A minor reduction of TH-immunopositive cells after aSyn(WT) expression was observed *in vitro*, but this loss was more pronounced after expression of aSyn(A30P) and aSyn(A53T). This is in line with previous findings on neuronal toxicity induced by aSyn(WT) and its mutant forms in primary mesencephalic cultures (Zhou et al., 2000), SH-SY5Y cells (Junn and Mouradian, 2002), drosophila neurons (Park et al., 2007), and more recently patient-derived induced pluripotent stem cells (Byers et al., 2011). Overexpression of all variants of aSyn principally can be neurotoxic for DAergic neurons and this can depend on the amount of protein (over-)expression and on the presence of additional stress factors. Importantly, this data also suggests that in addition to its pathogenic forms also aSyn(WT) can be toxic depending on the context. Although we did not observe a relevant cell loss in non-DAergic neurons, we found both a metabolic impairment and an increased cytotoxicity-related release of AK after expression of all aSyn variants. The culture duration may be too short to observe overt cell death in the non-dopaminergic sub-population, which appears less susceptible to aSyn overexpression than DAergic neurons.

These *in vitro* findings have been basically corroborated in different *in vivo* models of viral vector-mediated aSyn overexpression in the SNpc: AAV-mediated expression of human aSyn(WT) and aSyn(A53T) in rat nigral neurons resulted in a severe cellular pathology if the constructs were expressed under the strong chicken β-actin promoter. Here, loss of dopamine neurons accumulated to up to 80% in both aSyn(WT) and aSyn(A53T) forms, most likely because both forms of aSyn become toxic if expressed at higher levels (Kirik et al., 2002). Similar findings were obtained in a lentiviral-based model of phosphoglycerate kinase promoter-driven overexpression of human aSyn(WT), aSyn(A30P), and aSyn(A53T) in the SNpc of rats, where DAergic cell loss of about 30% after 5 months was observed for aSyn(WT) and aSyn(A30P) and to a lesser extent for aSyn(A53T) (Lo Bianco et al., 2002). In comparison, AAV-mediated nigral expression of the fibril forming variants of human aSyn(WT) and aSyn(A30P) both caused progressive DAergic cell loss but aSyn(WT) displayed the strongest cellular neurotoxicity in a different study. Here, the progressive cell loss was not found if prefibrillar and non-aggregating variants aSyn(A56P) and aSyn(A30P/A56P/A76P) were expressed because these forms rather induced acute DAergic cell death in the first 4 weeks (Taschenberger et al., 2012). In view of these data on DAergic survival, it seems that overexpression of human aSyn(WT) and both the aSyn(A30P) or aSyn(A53T) mutant forms is harmful and the extent of toxicity depends on the type of the viral vector and the promoter used.

In order to study neurotoxic effects of AAV-mediated nigral aSyn overexpression in the presence of an additional stressor we have employed the striatal 6-OHDA-lesion paradigm which by itself caused a DAergic cellular loss of about 30% being stable from 4 to 12 weeks after lesion. Here, aSyn overexpression caused a modest additional loss of TH-immunopositive neurons in the SNpc after 12 weeks, which was comparable for the aSyn WT, A30P, and A53T forms. Thus, aSyn overexpression appears to be only a mild neurotoxic factor *per se*, playing a less prominent role if a lesioning stressor, e.g., 6-OHDA, is already present. However, the mere DAergic neuronal survival does not indicate in how far their axonal projections can be preserved and if the intrinsic regenerative capacity of DAergic neurons is impaired. Therefore, we have analyzed the impact of aSyn overexpression on striatal axonal fiber loss and on the regenerative response both *in vitro* and *in vivo*.

The striatal 6-OHDA lesion itself caused a pronounced axonal degeneration and led to a decrease of striatal DAergic fiber density to 30% after 4 weeks, which recovered to 60% after 12 weeks in our model. Concomitant expression of aSyn(A30P) in the SNpc caused an additional reduction of TH-immunopositive striatal fibers 12 weeks after lesion, whereas there was a slight increase for aSyn(WT) and a non-significant effect for aSyn(A53T). This indicates that only the A30P variant impairs axonal stability under 6-OHDA stress conditions being in line with the aforementioned lentiviral study, in which the loss of striatal TH-immunopositive fibers was also most pronounced for aSyn(A30P) and less for aSyn(WT) or aSyn(A53T) (Lo Bianco et al., 2002). This observation was confirmed in aSyn(A30P) transgenic mice, in which the application of the mitochondrial complex I inhibitor MPTP strongly reduced the regeneration of DAergic fibers in comparison to wildtype animals. Interestingly, the DAergic degeneration in this model was further increased by chronic L-DOPA treatment, an oxidative stressor (Szegö et al., 2012). However, in this study the aSyn(A30P) transgenic mouse model was not compared with other variants of aSyn and the MPTP lesion does rather evoke a perikaryal lesion in contrast to 6-OHDA which causes a local lesion to the axonal compartment in the striatum.

In our comparative study, the negative impact of both aSyn mutants on neurite and axonal regeneration in DAergic cells was very prominent in comparison to the aSyn(WT) form. In the regeneration paradigm *in vitro*, the pathogenic mutant forms of aSyn reduced neurite regeneration by more than 30% whereas the wildtype form did not have a significant effect. In another study in SH-SY5Y cells neurite outgrowth was found to be modulated by human aSyn(WT) interacting with cytoskeletal proteins such as spectrin beta non-erythrocyte 1 (SPTBN1). Here, aSyn(WT) attenuated excess branching caused by SPTBN1 overexpression (Lee et al., 2012).

We observed an even stronger effect by overexpression of mutant aSyn on the level of striatal expression of the regeneration marker GAP-43. Here, aSyn(A30P) most prominently reduced GAP-43 expression followed by aSyn(A53T), whereas aSyn(WT) did not significantly affect GAP-43. To our knowledge, this is the first description of the effects of aSyn overexpression in DAergic cells on expression of the regeneration marker GAP-43. Other studies examining the rat 6-OHDA striatal lesion model found unchanged levels of striatal GAP-43 protein at 8 weeks after lesion, whereas there was a strong reduction in GAP-43-mRNA-immunopositive neurons in the SN which persisted over time (Iwata et al., 2001). Very interestingly, the neuropathological examination of a human PD patient who had been continuously infused with GDNF for 43 weeks into the right putamen revealed a clear increase in GAP-43 labeling at the ipsilateral side being associated with an improvement in the clinical UPDRS score and an increased ^18^F-dopa uptake in the ipsilateral posterior putamen (Love et al., 2005). This underlines the importance of GAP-43 as marker indicating regeneration in PD. Because striatal GAP-43 was shown to be significantly reduced by DAergic expression of aSyn(A30P) and aSyn(A53T) in our study, the mutant aSyn forms seem to be especially detrimental for the regenerative response.

The A30P form has also been shown to provoke cytoskeletal alterations such as an increase in levels of filamentous actin (Chung et al., 2009). In line with this, a cell culture study on hippocampal neurons with aSyn(A30P) displayed distinct effects from aSyn(WT) and increased the rate of actin polymerization with subsequent disruption of the cytoskeleton and induction of actin-rich foci (Sousa et al., 2009). This is indicative of a decreased remodeling of axonal terminals and reduced axonal outgrowth capacity which is similarly seen when there is an increased activity of the Rho/ROCK signaling pathway (Tönges et al., 2011a). We have examined the activation of the Rho/ROCK in DAergic cultures *in vitro* and could observe a strong activation by both aSyn mutants which was not the case for aSyn(WT). As pharmacological ROCK-inhibition has recently been demonstrated to be a successful strategy to foster axonal regeneration in toxin-based models of PD (Tönges et al., 2012), this approach could also be worthwhile to be evaluated in aSyn-related genetic models of PD.

Our findings indicate that genetic alterations of aSyn have a clear impact on the regenerative capacity of DAergic neurons both *in vitro* and *in vivo*. In our model systems, aSyn(A53T) and, more importantly aSyn(A30P), impaired neurite regeneration of MDN *in vitro* and reduced TH-immunopositive axonal fiber sprouting of 6-OHDA lesioned DAergic neurons *in vivo*. This broadens our understanding of pathogenic effects of aSyn and its disease-relevant mutants beyond DAergic neurotoxicity. Accumulating data now suggest an involvement of the ROCK signaling pathway in the pathophysiology of preclinical PD models. This is a strong argument for the examination of its contribution also to human PD because a variety of ROCK targeting strategies are becoming available in the clinics and could easily applied in a translational approach for this debilitating disease.

### Conflict of interest statement

The authors declare that the research was conducted in the absence of any commercial or financial relationships that could be construed as a potential conflict of interest.

## Abbreviations

AAV: adeno-associated virus
aSyn: alpha-synuclein
ANOVA: analysis of variance
BSA: bovine serum albumin
CBA: chicken β-actin
DA: dopamine
DAergic: dopaminergic
DAPI: 4,6-diamidino-2-phenylindole
DIV: day *in vitro*
DMEM: Dulbecco's modified Eagle's medium
GAP-43: growth associated protein 43
GDNF: Glial cell line-derived neurotrophic factor
MDN: midbrain dopaminergic neurons
PBS: phosphate-buffered saline
PFA: paraformaldehyde
PD: Parkinson's disease
ROCK: rho kinase
RT: room temperature
SNpc: Substantia nigra, pars compacta
TH: tyrosine hydroxylase
WT: wildtype.
