# Supplementary material for: Alpha-synuclein mutations impair axonal regeneration in models of Parkinson's disease
Source: Front Aging Neurosci. 2014 Sep 10;6:239. doi: 10.3389/fnagi.2014.00239 (PMC4159996; doi:10.3389/fnagi.2014.00239)
Supplement: Supplementary file 1 [file DataSheet2.PDF]

Tönges el al.: Alpha-synuclein mutations impair axonal regeneration in models of Parkinson´s disease

Supplementary Data

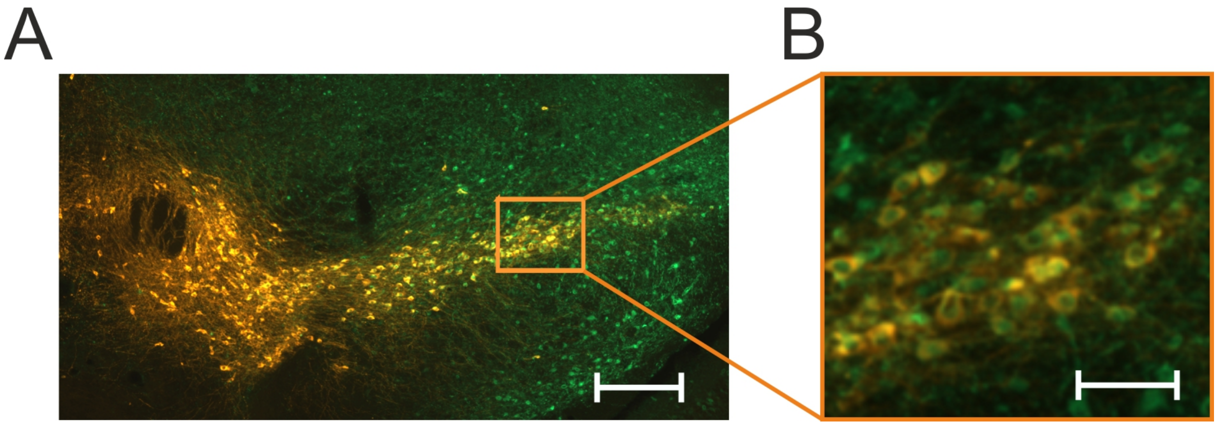

Suppl. Fig. 1: Immunohistochemical detection of AAV6 expressing EGFP/human aSyn (1x10^8 transducing units) four weeks after stereotactical nigral injection. Overlays of representative micrographs of substantia nigra mouse brain sections with intrinsic EGFP expression (green) and imunolabeling against TH (Cy3, red). Scale bar = 250 µm (left) and 50 µm (right).

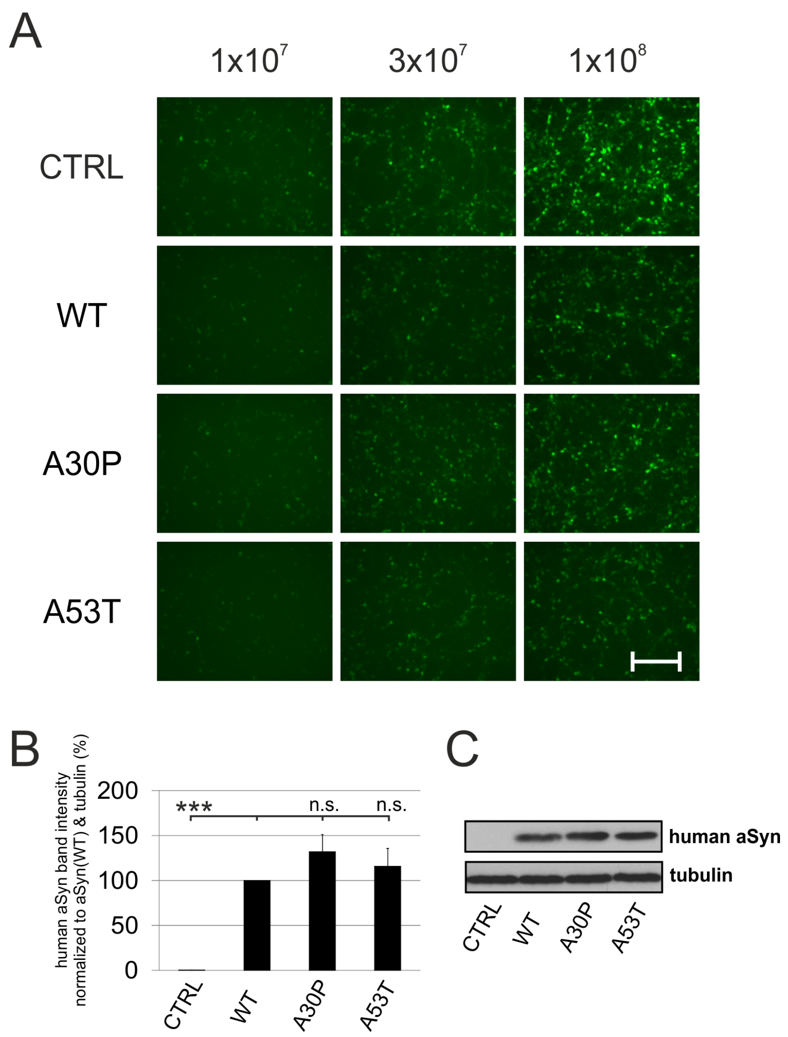

Suppl. Fig. 2: AAV constructs expressing EGFP or human aSyn/EGFP in MDN after 6 days in culture. (A) Micrographs of EGFP-immunofluorescence in MDN cultures which have been infected with AAV constructs ($1x10^7$, $3x10^7$ or $1x10^8$ transducing units) expressing EGFP (CTRL) or variants of aSyn (WT, A30P or A53T) and EGFP. Scale bar 200 µm. (B) Quantification of human aSyn normalized to aSyn(WT) and tubulin in MDN cultures infected with each 1×10^8 transducing units. (C) Representative immunoblots of human aSyn and tubulin.

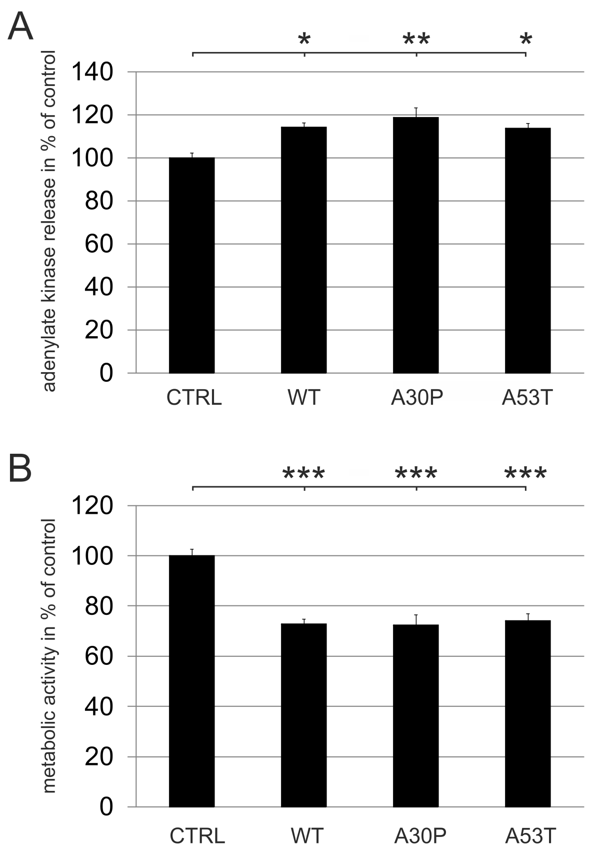

Suppl. Fig. 3: Cell toxicity (ToxiLight^{TM}) and cell viability (WST-1) assays of primary MDN at DIV6. (A) Histogram showing relative cell toxicity based on the release of adenylate kinase in MDN transduced with AAV expressing human aSyn (WT, A30P or A53T; each $1x10^8$ transducing units) compared to AAV expressing EGFP ($1x10^8$ transducing units). (B) Histogram showing relative metabolic activity in MDN treated as in (A). Bars represent means ± SEM. *p<0.05, *p<0.01, ***p<0.001.

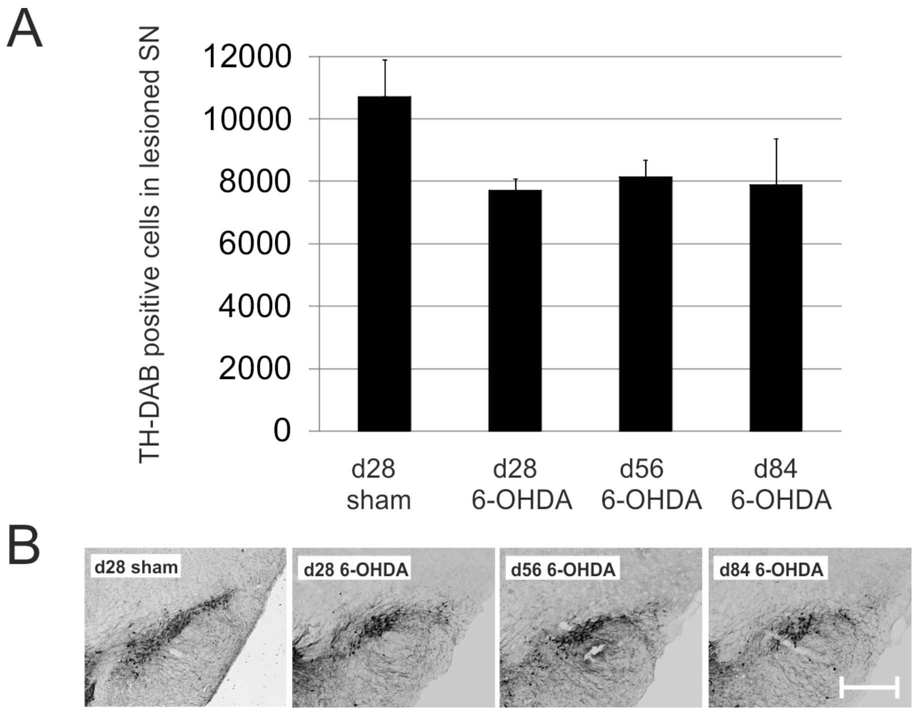

Suppl. Fig. 4: DAergic cell survival in the SN at different time points after unilateral striatal 6-OHDA lesion. Striatal lesions were performed unilaterally with 6-OHDA (4 µg/2ul) or PBS (sham) and mice were analyzed at the indicated time points. (A) Histogram showing numbers of surviving TH-immunopositive nigral neurons at different time points after lesion. (B) Representative micrographs of substantia nigra mouse brain sections labeled against TH (black). Bars represent means ± SEM. Scale bar = 500 µm.

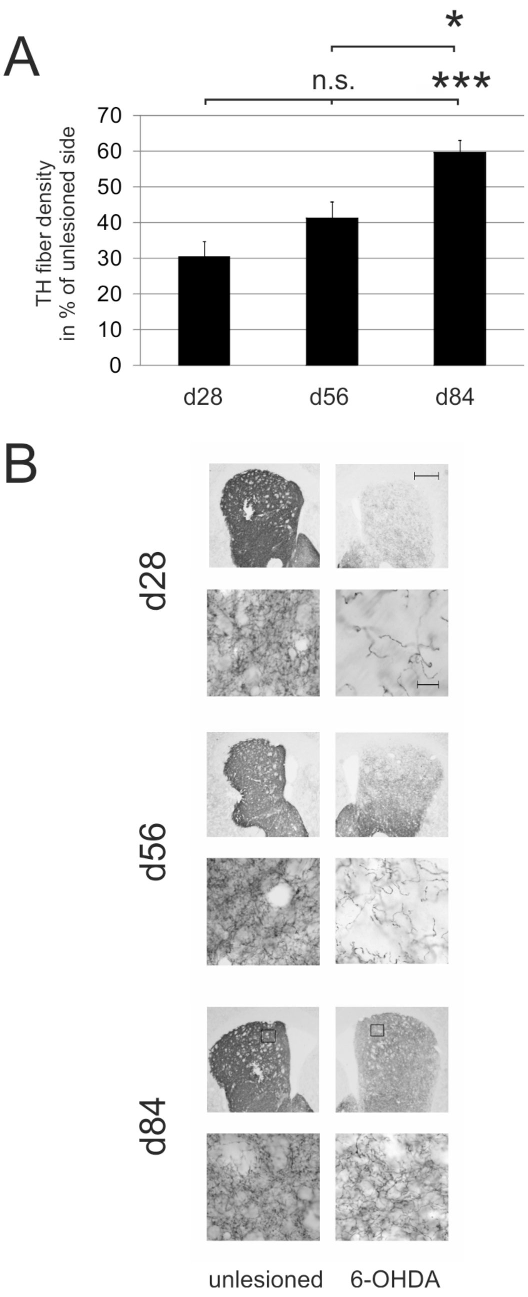

Suppl. Fig. 5: Density of TH-immunopositive striatal fibers twelve weeks after ipsilateral striatal 6-OHDA lesion. Nigral neurons were lesioned in the ipsilateral striatum with 6-OHDA (4 µg/2ul). (A) Histogram showing the relative density of TH-immunopositive striatal fibers in comparison to the unlesioned striatum twelve weeks after lesion vector injection. Bars represent means ± SEM. n.s. = not significant; *p<0.05, ***p<0.001. (B) Representative micrographs of the striatum and magnifications after immunostaining for TH (black). Black squares mark exemplary fields of fiber analysis. Bars represent means ± SEM. Scale bar = 500 µm (overview), 20 µm (magnification).

Supplementary Table 1

Survival of non-dopaminergic cells after AAV-mediated aSyn expression in vitro

**AAV construct**	**Surviving non-dopaminergic cells**
CTRL	100.00 ± 3.57 %
WT	101.74 ± 1.03 %
A30P	99.32 ± 4.25 %
A53T	98.54 ± 0.90 %

Supplementary Table 2

Survival of TH-immunopositive dopaminergic cells at the scratch border

AAV construct	Surviving dopaminergic cells
CTRL	100.00 ± 13.07 %
WT	86.26 ± 7.15 %
A30P	69.43 ± 10.11 %
A53T	61.10 ± 6.46 %
